# Hypertensive crisis and cheese

**DOI:** 10.4103/0019-5545.44910

**Published:** 2009

**Authors:** T. S. Sathyanarayana Rao, Vikram K. Yeragani

**Affiliations:** Department of Psychiatry, JSS University, JSS Medical College Hospital, Mysore - 570004, India; 1 Department of Psychiatry and Behavioral Neurosciences, Wayne State University, Detroit, MI, USA; 1 Visiting Professor of Psychiatry, University of Alberta, Edmonton, Canada; 1 Visiting Professor of Biotechnology, Aacharya Nagarjuna University, A.P., India

**[Editor's Note:** This article is one of the new Sections we are adding to encourage the psychiatric trainees and other faculty members to critically examine some of the interesting discoveries and phenomena that relate to Psychiatry directly or indirectly. Many great discoveries have been rather serendipitous experiences in Medicine, in general. We would like to begin this with a very significant side-effect of monoamine oxidase inhibitors (MAOI), the “cheese reaction”. These drugs came into psychiatric practice since the euphoriant effects of ipronizid were uncovered during treatment of tuberculosis.]

“*It is not enough to know that cheese is a bad article of food for cheese does not harm all people equally. If it had been harmful to the human constitution in general, everyone would have suffered. Now whoever knows these facts will not suffer*”

*HIPPOCRATES: GREEK MEDICINE*

The tyramine connection was discovered by a British pharmacist whose wife was taking an monoamine oxidase inhibitors (MAOI). He noticed that every time they had a meal with cheese, she would get a severe headache. Cheese, especially aged cheese, contains substantial amount of tyramine. For this reason, persons taking MAOI antidepressants are cautioned to avoid foods that are rich in tyramine so that the hypertensive crises can be avoided. However, the road to understanding the neurochemical mechanism of this phenomenon was rather a long and tortuous one. Hypertensive crises due to phenelzine were reported by Dally and Tailor in 1962 but these episodes are commoner with tranylcypromine. Barry Blackwell systematically described these side-effects in 12 patients and 10 of these were women. Eleven patients were receiving tranylcypromine and one, phenelzine. In majority that had the reaction, cooked or raw cheese was the precipitating agent. Increases in blood pressure (BP) ranged from 160/90 to 220/115 mm Hg. The onset of the episode was usually one to two hours after the food intake. Headache was the main symptom associated with heart pounding and palpitations and the complications included subarachnoid hemorrhage, hemiplegia, intracranial hemorrhage, cardiac arrhythmias, cardiac failure, pulmonary edema, and death.

Dally noted the striking similarity of this phenomenon to symptoms of pheochromocytoma and suggested that the release of pressor amine locally or systemically might be responsible for this effect. The increase in tissue concentrations of epinephrine and norepinephrine supports this view. Cheese contains both tyramine and histamine. Tyramine was the first known substrate of monoamine oxidase (MAO). Tyramine was first isolated from cheese and later named after the Greek (tyros) for cheese. Tyramine oxidase occurs in high concentrations in intestinal mucosa. The pressor amines in the gut form as a result of bacterial decarboxylation of amino acids. In this context, it should be noted that the adverse effects resulting from the absorption of some of these amines was described by Metchinkoff as early as in 1905. Blackwell and co-workers have done substantial amount of work on this issue along with many other great researchers. During ripening of cheeses, the casein is broken down to form peptides and free aminoacids. In 1965[1] in their seminal article, Blackwell and co-workers have shown that tyramine in cheese is related to the hypertensive crises after MAO inhibition. They have determined the tyramine content of 14 different cheddar cheeses and several Wensleydale, Cheshire and Caerphilly cheeses. The reactions were variable as the amount of tyramine in the 14 cheddar cheeses varied from 72 to 953 mcg per gram of cheese. An oral dose of even 6 mg of tyramine can increase BP. However, the dangerous dose for different people varies significantly from anywhere about 25 mg of tyramine. Thus, Blackwell and co-workers[1–3] were the first to systematically quantify the amines in cheese.

In addition to tyramine, hypertensive responses can also be associated particularly with over-the-counter sympathomimetic drugs such as ephedrine, pseudoephedrine and phenylpropanolamine, which are present in several decongestants and cough medicines. Hypertension may also occur when MAOIs are combined with L-dopa, methylphenidate, dextroamphetamine, reserpine, guanethidine, or tetrabenazine. Deprenyl, a specific MAO-B inhibitor at low doses (10 mg/day), can be administered safely with dietary tyramine, L-dopa, or L-dopa plus a decarboxylase inhibitor. For clinicians, the differentiation of true hypertensive crises from rebound headaches caused by MAOI-induced postural hypotension is important to treat the hypertensive crises early. Agents normally used to lower blood pressure during a hypertensive crisis include nifedipine, a calcium channel blocker or phentolamine, an alpha adrenergic blocker.
